# Mesenchymal Stromal Cell Spheroids as an Emerging Regenerative Strategy for Osteonecrosis of the Femoral Head

**DOI:** 10.3390/cells15191787

**Published:** 2026-09-30

**Authors:** Sholpan Mukhlis, Meruyert Imanbekova, Dina Saginova, Madina Sarsenova, Aida Nurgaliyeva, Nursulu Altaeva, Vyacheslav Ogay

**Affiliations:** 1 Stem Cell Laboratory, National Center for Biotechnology, 010000 Astana, Kazakhstan; muhlis@biocenter.kz (S.M.); meruert.iman@gmail.com (M.I.); sarsenova@biocenter.kz (M.S.); aida.nur.kaz@gmail.com (A.N.); 2 National Scientific Center for Traumatology and Orthopedics After Academician N.D. Batpenov, 010000 Astana, Kazakhstan; sa_dina@mail.ru; 3 Department of Medical Genetics and Molecular Biology, Astana Medical University, 010000 Astana, Kazakhstan; altaeva.n@amu.kz

**Keywords:** osteonecrosis of the femoral head, mesenchymal stromal cells, MSC spheroids, angiogenesis, osteogenesis, regenerative medicine, cell therapy

## Abstract

Osteonecrosis of the femoral head (ONFH) is a progressive ischemic bone disorder that may lead to subchondral fracture, femoral-head collapse, and total hip arthroplasty. Impaired regeneration reflects persistent ischemia, endothelial dysfunction, defective angiogenesis and bone remodeling, inflammation, oxidative stress, and dysfunction of endogenous mesenchymal stromal cells (MSCs). Although MSC therapy, particularly with core decompression in precollapse ONFH, is a promising joint-preserving approach, conventional two-dimensional (2D)-expanded MSCs are limited by poor survival and retention, anoikis, disrupted cell–cell and cell–extracellular matrix interactions, and loss of potency during ex vivo expansion. Three-dimensional MSC spheroids may overcome several of these limitations by preserving intercellular and matrix interactions and enhancing resistance to ischemic stress, paracrine signaling, angiogenic activity, immunomodulation, and osteogenic competence. Hypoxia-responsive signaling, autophagy, trophic-factor secretion, and extracellular vesicle-mediated communication may promote angiogenesis–osteogenesis coupling and the restoration of the ischemic bone–vascular niche. This review summarizes the pathophysiological barriers to ONFH regeneration, current evidence and limitations of conventional MSC therapy, biological mechanisms underlying MSC-spheroid activity, and emerging strategies for therapeutic optimization. Importantly, direct evidence demonstrating the superiority of MSC spheroids over conventional MSC preparations specifically in ONFH remains limited. Future translation will require disease-specific comparative studies, standardized GMP-compatible manufacturing, validated potency assays and clinically practical delivery systems.

## 1. Introduction

Osteonecrosis of the femoral head (ONFH), or avascular necrosis, is a progressive skeletal disorder characterized by impaired blood supply, death of osteocytes and bone marrow cells, and deterioration of subchondral architecture [1,2,3]. It predominantly affects young and middle-aged adults and may progress to subchondral fracture, femoral-head collapse, secondary osteoarthritis, and total hip arthroplasty (THA). Because many affected patients are relatively young, the preservation of the native femoral head before structural collapse remains a major therapeutic objective [1,4]. ONFH is multifactorial, involving vascular injury, ischemia, endothelial dysfunction, abnormal bone remodeling, oxidative stress, inflammation, and impaired endogenous mesenchymal stromal cell (MSC) function [1,2,3]. Together, these processes disrupt the bone–vascular regenerative niche and progressively weaken the trabecular network.

Disease stage strongly influences therapeutic outcomes. Once substantial collapse and secondary degeneration have developed, THA remains the most predictable treatment, whereas precollapse and early pericollapse disease provide a therapeutic window for joint-preserving interventions [1,4]. Core decompression (CD) can reduce intraosseous pressure and facilitate tissue ingrowth but does not reliably restore the vascular and cellular components lost during osteonecrosis, particularly in larger lesions [1,2,4].

MSC therapy has therefore attracted considerable interest because MSCs combine osteogenic potential with paracrine, proangiogenic, immunomodulatory, and cytoprotective properties. Clinical studies and meta-analyses suggest that MSC augmentation of CD may improve femoral-head preservation in selected patients with early-stage ONFH [5,6,7,8], although outcomes remain heterogeneous because of differences in patient selection, lesion characteristics, cell source and dose, manufacturing, delivery, and follow-up [5,6].

Conventional two-dimensional (2D)-expanded MSCs have important limitations. Enzymatic detachment disrupts cell–cell and cell–extracellular matrix (ECM) interactions, and dissociated cells implanted into osteonecrotic tissue encounter hypoxia, nutrient deprivation, oxidative stress, inflammation, and limited matrix anchorage. These conditions can promote apoptosis and anoikis, reduce retention, and shorten therapeutic activity [9]. Three-dimensional MSC spheroids have emerged as a potential strategy to address some of these limitations by preserving intercellular contacts and endogenous ECM [9,10]. Experimental studies indicate that spheroid formation can alter survival, secretory activity, immunomodulation, and osteogenic competence, but these effects depend on cell source, aggregate size, culture conditions, and manufacturing method [10,11,12,13,14,15].

The potential relevance of MSC spheroids to ONFH lies in their ability to support several functions required for regeneration of the ischemic bone–vascular niche. However, most evidence for spheroid-associated benefits derives from bone-defect, ischemic-tissue, inflammatory, and vascularized tissue-regeneration models rather than ONFH itself [14,15,16]. These studies provide mechanistic and proof-of-concept evidence but do not establish disease-specific superiority. Direct head-to-head comparisons in ONFH remain limited.

Accordingly, this review critically examines MSC spheroids as an emerging regenerative strategy for ONFH. We address the pathological and biomechanical barriers to regeneration, current evidence and limitations of conventional MSC therapy, the biological consequences of three-dimensional MSC organization, and the distinction between direct ONFH evidence and indirect supporting data. We also discuss therapeutic optimization, competing regenerative approaches, biomaterial and biomechanical considerations, and the manufacturing, safety, regulatory, and translational requirements for clinical development.

### Literature Search Strategy

A literature search was conducted in PubMed, Scopus, and Web of Science to identify studies relevant to MSC-based and MSC-spheroid-based approaches for ONFH. The search was performed through July 2026 without a predefined lower publication-date restriction; the studies included in the final reference set were published between 2008 and 2026. Search terms included combinations of “osteonecrosis of the femoral head”, “femoral head osteonecrosis”, “avascular necrosis”, “mesenchymal stromal cells”, “mesenchymal stem cells”, “MSC spheroids”, “3D MSC culture”, “three-dimensional cell aggregates”, “angiogenesis”, “osteogenesis”, “hypoxia”, “extracellular vesicles”, “biomaterials”, and “bone regeneration”. Additional relevant studies were identified by screening the reference lists of selected articles. Original preclinical and clinical studies, systematic reviews, meta-analyses, and selected mechanistic studies were considered when relevant to ONFH pathophysiology, MSC therapy, spheroid biology, or regenerative mechanisms potentially applicable to ONFH. Particular attention was given to distinguishing direct ONFH evidence from indirect mechanistic evidence derived from bone-defect, ischemic-tissue, and other regenerative models. Study selection was based on relevance to the scope of this narrative review, and no formal meta-analysis was performed.

## 2. Pathophysiological Barriers to Regeneration in ONFH

ONFH represents progressive failure of the bone–vascular regenerative niche rather than an isolated ischemic event. Persistent ischemia and endothelial dysfunction, defective trabecular remodeling, inflammation and oxidative stress, and dysfunction of endogenous MSCs interact to limit revascularization, replacement of necrotic bone, and maintenance of mechanical integrity [1,2]. These barriers are also relevant to cell therapy because transplanted cells must remain functional within the same hostile microenvironment.

### 2.1. Ischemic and Hypoxic Microenvironment

The disruption of femoral-head perfusion is the initiating event in ONFH. Traumatic vascular interruption and non-traumatic mechanisms, including endothelial injury, thrombosis, fat embolism, venous stasis and increased intraosseous pressure can reduce microvascular flow below that required for tissue viability [1,17]. Endothelial dysfunction may persist after the initial insult. In glucocorticoid-induced ONFH, patient-derived bone microvascular endothelial cells show reduced viability, migration, and tube formation with increased apoptosis [18].

Hypoxia initially activates compensatory pathways, but persistent vascular injury limits effective angiogenesis. Prolonged oxygen deprivation contributes to mitochondrial dysfunction, oxidative stress, senescence and loss of osteogenic function [19,20]. Thus, the necrotic region combines a strong demand for vascular regeneration with impaired endothelial and progenitor-cell function.

For regenerative therapy, the relevant distinction is between adaptive hypoxic signaling and prolonged severe ischemia. Transplanted MSCs must survive the early ischemic period long enough to support revascularization before nutrient and oxygen deprivation eliminate therapeutic activity.

### 2.2. Disruption of the Trabecular-Vascular Architecture and Defective Bone Remodeling

Femoral-head integrity depends on the coupled organization of trabecular bone and the intraosseous vascular network. Following ischemic injury, osteocyte death precedes overt structural collapse. Reparative activity is predominantly localized to the viable-necrotic interface, where osteoclastic resorption of necrotic bone occurs concurrently with new-bone formation. When removal of necrotic bone exceeds formation of mechanically competent new bone, structural weakness increases [1,2].

Human micro-CT and biomechanical studies demonstrate reduced bone mineral density, trabecular thickness and number, microfractures, increased osteoclastic activity and impaired mechanical properties within necrotic and subchondral regions [21,22]. Accordingly, the preservation of gross femoral-head contour during early ONFH does not imply preserved biomechanical competence, and subchondral fracture is closely associated with regions of active bone resorption.

Vascular and trabecular deterioration are mutually reinforcing: the loss of vessels restricts oxygen, nutrients, and osteogenic signals, whereas inadequate trabecular reconstruction limits the framework for vascular ingrowth. Durable repair therefore requires the coordinated restoration of vascular supply and mechanically competent bone.

### 2.3. Chronic Inflammatory Signaling and Oxidative Stress

Sterile inflammation and oxidative stress further impair the regenerative niche. Necrotic tissue activates immune cells, and persistent M1-like macrophage responses, can increase TNF-α, IL-1β, and IL-6, promote osteoclastogenesis, and suppress osteogenic activity. In glucocorticoid-associated ONFH, M1 macrophage-conditioned medium induced the apoptosis of MSCs and osteocytes through TNF-α/NF-κB signaling, whereas early M1 depletion slowed disease progression [23].

Inflammatory signaling also favors RANKL-dependent bone resorption and can impair endothelial function. In parallel, glucocorticoids, ischemia-reperfusion, mitochondrial dysfunction, and lipid accumulation increase reactive oxygen species (ROS), which damage cellular macromolecules, promote apoptosis or senescence, and suppress osteogenic and angiogenic activity [3,17,24].

These processes form a reciprocal amplification loop that makes the necrotic region unfavorable for regeneration. Effective MSC-based therapy must therefore address not only cell number but also the inflammatory and oxidative environment that limits cell survival and function.

### 2.4. Reduced Endogenous Regenerative Potential of Mesenchymal Progenitor Cells

Endogenous regenerative failure also reflects the dysfunction of the local MSC compartment. Glucocorticoid exposure and excessive alcohol consumption can shift BM-MSC differentiation from osteogenic toward adipogenic programs, reducing osteoblast formation and adversely affecting the marrow microenvironment [3].

Patient-derived evidence confirms broader cellular dysfunction. BM-MSCs isolated from necrotic femoral-head regions show impaired osteogenesis, enhanced adipogenesis, mitochondrial dysfunction, oxidative stress and senescence [25]. Human ONFH specimens also contain cells expressing senescence-associated markers, including p16 (INK4a), p21, and p53, suggesting that cellular senescence may further compromise local tissue repair by reducing regenerative capacity and promoting pro-inflammatory signaling [26].

Thus, the transplantation of healthy MSCs does not by itself remove the pathological host environment. Exogenous cells may acquire similar deficits unless their stress resistance and the surrounding niche are simultaneously improved, providing a rationale for optimized cell configurations and supportive delivery systems.

### 2.5. Convergence of Biological and Mechanical Failure

**Figure 1:**
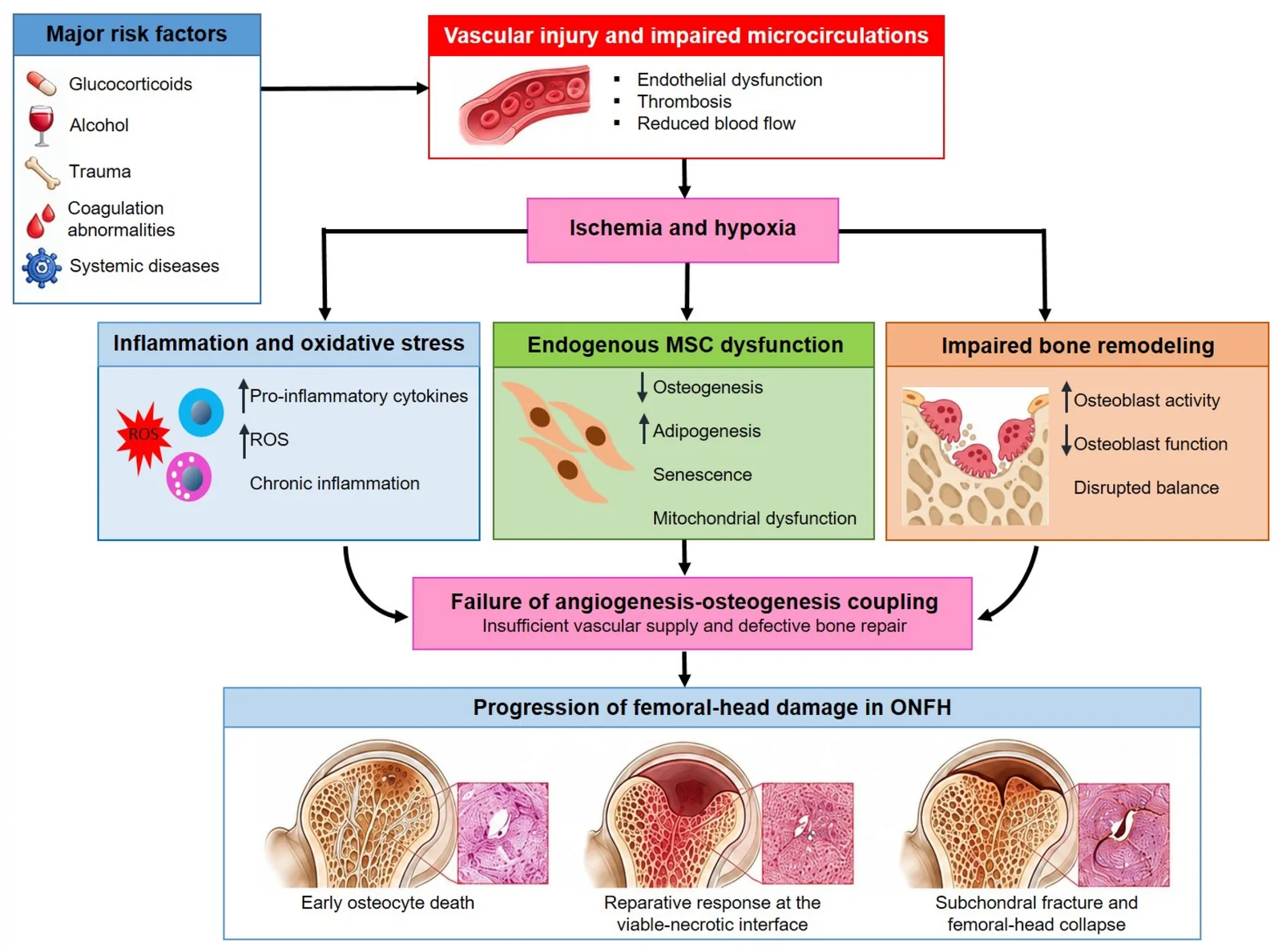
Pathophysiological barriers to regeneration in ONFH and their contribution to failure of angiogenesis–osteogenesis coupling. Major risk factors, including glucocorticoid exposure, excessive alcohol consumption, trauma, coagulation abnormalities, and systemic diseases, can initiate vascular injury characterized by endothelial dysfunction, thrombosis, and reduced intraosseous blood flow, resulting in persistent ischemia and hypoxia [1,2,3,17,18]. The ischemic microenvironment promotes inflammatory and oxidative stress, including increased pro-inflammatory cytokines and ROS, while impairing endogenous MSC function through reduced osteogenesis, increased adipogenesis, cellular senescence, and mitochondrial dysfunction [23,24,25,26]. In parallel, increased osteoclastic activity and impaired osteoblast function disrupt bone remodeling, limiting replacement of necrotic trabeculae with mechanically competent new bone [21,22]. These interconnected processes converge on failure of angiogenesis–osteogenesis coupling, resulting in insufficient vascular supply and defective bone repair. The lower sequence illustrates disease progression from early osteocyte death through reparative activity at the viable–necrotic interface to progressive trabecular weakening, subchondral fracture, and femoral-head collapse. Black arrows indicate causal or reinforcing relationships among pathological processes, whereas ↑ and ↓ indicate increased and decreased biological responses, respectively.

The principal barriers to ONFH regeneration are interconnected rather than independent. As summarized in Figure 1, vascular injury and persistent ischemia, inflammation and oxidative stress, endogenous MSC dysfunction, and impaired bone remodeling converge on failure of angiogenesis–osteogenesis coupling and ultimately compromise femoral-head structural integrity.

These interacting abnormalities create a mismatch between vascular recovery and skeletal repair. Even when reparative activity occurs at the viable–necrotic interface, the resorption of necrotic trabeculae may outpace the formation of mechanically competent new bone. Continued physiological loading then promotes microfracture formation within the structurally weakened subchondral region [21,22,27].

Once subchondral fracture develops, biological repair must occur within a structure that is progressively losing its load-bearing capacity. This helps explain why regenerative approaches are most plausible in precollapse disease, when sufficient architecture remains to support vascular and bone reconstruction. Effective therapy must therefore combine biological regeneration with preservation of mechanical integrity.

## 3. Mesenchymal Stromal Cell Therapy for ONFH: Current Evidence and Limitations

MSC therapy is being investigated as a biological adjunct for joint preservation in ONFH, particularly during precollapse disease. Most clinical approaches combine MSC delivery with CD, aiming to supplement osteogenic capacity while modulating the ischemic bone–vascular microenvironment [28].

### 3.1. Sources of MSCs Used for ONFH Therapy

BM-MSCs are the most extensively studied source and remain the clinical benchmark. Cultured autologous BM-MSCs combined with CD have shown favorable long-term outcomes in selected early-stage cohorts [7,29], whereas BMAC contains heterogeneous cell populations and relatively few true MSCs; this difference may contribute to inconsistent results, including the neutral randomized findings of Pepke et al. [30].

AD-MSCs are readily accessible and proliferative, but clinical evidence remains limited. Yoon et al. [31] reported increased vascularity in 79% of evaluable hips after local AD-MSC implantation without a significant reduction in lesion size or improvement in major functional scores. UC-MSCs offer an allogeneic, bankable source; a small intra-arterial study reported improved perfusion and reduced necrotic volume [32], and preclinical data support proangiogenic activity [33].

Other sources, including synovial-fluid and dental-pulp stromal cells, remain predominantly preclinical [34,35]. Overall, differences in tissue source, product composition, expansion history and dose complicate direct comparison among MSC preparations.

### 3.2. Routes and Strategies for MSC Delivery

Delivery strongly influences retention and target-site exposure. Local intraosseous transplantation after CD provides direct access to the necrotic region and is the most established approach [29,36], whereas intra-arterial delivery is less invasive but depends on cell homing and provides less control over local dose [32].

Biomaterial-assisted delivery can improve retention and provide matrix-like or microenvironmental support. Experimental ONFH systems incorporating MSCs, endothelial progenitor cells or oxygen-releasing components have improved graft survival and regenerative outcomes [37,38,39]. However, the contribution of the biomaterial may be difficult to separate from that of the cells.

### 3.3. Preclinical Evidence

Preclinical ONFH studies generally report improvements in bone formation, trabecular architecture, vascularization, or femoral-head preservation after MSC-based interventions [33,34,35,37,38,39,40]. The strongest responses often occur when vascular and skeletal repair are targeted together, for example through endothelial co-transplantation, oxygen-generating systems, or bioactive scaffolds [38,39]. These findings support a coupled vascular–osteogenic strategy but remain dependent on the animal model, product and delivery platform.

### 3.4. Clinical Studies of MSC Therapy

Clinical studies have predominantly evaluated autologous BM-MSCs in combination with core decompression. Overall, the available evidence indicates an acceptable short- to medium-term safety profile and suggests that cell-based augmentation may improve joint preservation in selected patients with precollapse ONFH. However, the magnitude and consistency of benefit vary substantially among studies, indicating that MSC therapy should not be regarded as a uniform intervention.

An important source of heterogeneity is patient selection and disease severity. The most favorable outcomes have generally been reported in precollapse disease, when the subchondral architecture remains mechanically salvageable. In contrast, larger lesions, extensive involvement of the load-bearing region, and established structural compromise reduce the likelihood that a biological intervention can restore sufficient mechanically competent bone before collapse occurs. This stage dependence is illustrated by Kang et al. [36], who reported a lower rate of conversion to THA after BM-MSC implantation than after core decompression alone (28.3% vs. 49%), with the strongest benefit in ARCO I–II disease; however, overall ARCO-stage progression was not significantly prevented.

Lesion burden may also explain discrepancies between biological and clinical endpoints. Yoon et al. [31] observed increased vascularity in 79% of evaluable hips after AD-MSC implantation, yet neither necrotic-lesion size nor major functional scores improved significantly. Thus, enhancement of perfusion does not necessarily translate into structural recovery when the amount of necrotic bone exceeds the regenerative capacity of the intervention.

Cell-product characteristics represent another major source of variability. BMAC, culture-expanded BM-MSCs, AD-MSCs, and UC-MSCs differ substantially in cellular composition, expansion history, paracrine profile, and administered MSC dose. BMAC contains a heterogeneous mixture of hematopoietic, endothelial, stromal, and other cells, with only a relatively small MSC fraction, whereas culture-expanded products allow administration of a more defined cell population. This distinction may contribute to the neutral randomized findings of Pepke et al. [30], in which BMAC did not significantly improve clinical outcome, lesion volume, or femoral-head survival compared with core decompression alone. Moreover, administered doses vary by orders of magnitude among studies, from 2 × 10^6^ cultured BM-MSCs in Zhao et al. [29] to 1 × 10^8^ AD-MSCs in Yoon et al. [31] and 140 × 10^6^ BM-MSCs in the ORTHO2 study [7], and an optimal dose has not been established.

Delivery strategy and follow-up duration further complicate comparison. Local intraosseous implantation after core decompression provides direct access to the necrotic lesion but makes it difficult to separate the biological contribution of the cells from that of the surgical procedure. Intra-arterial administration is less invasive but relies on cell homing and provides less control over the number of cells reaching the femoral head. Similarly, short-term studies may detect early improvements in pain, perfusion, or imaging parameters without establishing durable preservation of femoral-head architecture, whereas longer follow-up is required to assess clinically meaningful endpoints such as structural progression and THA conversion.

A recent network meta-analysis including 29 comparative studies and 2177 hips reported favorable effects of core decompression combined with cell-based therapy on structural progression but did not demonstrate a significant reduction in conversion to THA [41]. Taken together, the available clinical evidence suggests that treatment response depends on the interaction among disease stage, lesion burden, cell source and dose, manufacturing procedure, delivery route, concomitant surgery, and follow-up duration. Representative quantitative outcomes from preclinical and clinical studies of MSC-based therapy for ONFH are summarized in Table 1. Future trials should therefore use standardized cell products, prospectively stratify patients by disease stage and lesion characteristics, and apply harmonized structural and clinical endpoints.

### 3.5. Major Limitations of Conventional 2D-Expanded MSCs

Conventional 2D-expanded MSC therapy is limited by poor post-transplantation survival and retention. Enzymatic detachment disrupts cell–cell and cell–ECM interactions and can increase susceptibility to anoikis and apoptosis [42], while single-cell suspensions may be rapidly lost from the target lesion. These problems are amplified in ONFH by ischemia, nutrient deprivation, oxidative stress, inflammation, and impaired vascularization [39].

Obtaining clinically relevant cell numbers also requires ex vivo expansion. Repeated passaging can promote replicative senescence, DNA damage, reduced proliferation, altered gene expression, and the loss of differentiation potential [43,44]. Donor, tissue source, passage number, culture conditions, and expansion history therefore contribute to product variability and may reduce functional potency despite preserved conventional identity markers.

Together, limited retention, sensitivity to the hostile ONFH microenvironment, functional deterioration during expansion and batch-to-batch variability constrain conventional 2D-expanded MSC therapy. These limitations motivate alternative cellular configurations and delivery strategies.

## 4. Mesenchymal Stromal Cell Spheroids for the Treatment of ONFH

MSC spheroids have been developed as an alternative cellular configuration intended to improve cell survival and preserve regenerative function after expansion. By organizing MSCs into three-dimensional aggregates, spheroids can modify cellular metabolism, survival, secretory activity, and osteogenic behavior [45]. Their effects, however, depend on product design and should not be assumed to be uniformly advantageous.

### 4.1. Biological Features of MSC Spheroids Compared with Conventional 2D-Expanded MSCs

During conventional monolayer expansion, MSCs adhere to rigid plastic substrates and adopt a flattened morphology that differs substantially from their organization within native tissues. Enzymatic detachment for transplantation further disrupts cell–cell and cell–ECM interactions. In contrast, spheroid formation promotes cellular condensation, extensive intercellular contact, and retention of endogenous ECM, thereby creating a three-dimensional microenvironment with distinct structural and biochemical characteristics [46].

**Figure 2:**
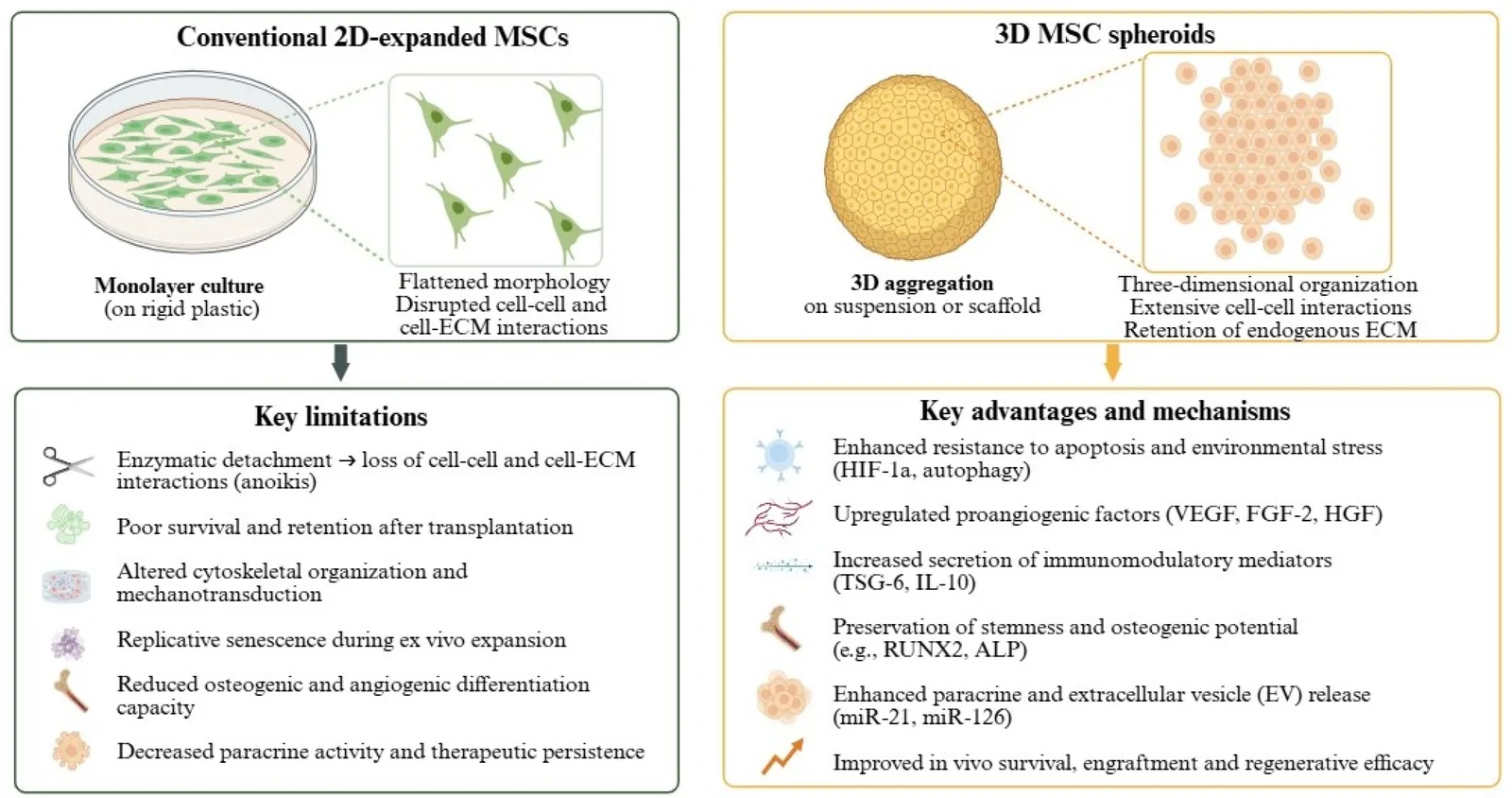
Comparison of conventional 2D-expanded MSCs and 3D MSC spheroids. Conventional monolayer expansion on rigid plastic limits cell–cell and cell–ECM interactions, while enzymatic detachment disrupts these interactions and may contribute to anoikis, reduced post-transplantation survival and retention, cellular senescence, and loss of therapeutic potency [42,43,44]. In contrast, 3D spheroid culture promotes extensive cell–cell interactions and retention of endogenous ECM [11,46], supports adaptive hypoxic and autophagic responses [12,47], and enhances secretion of proangiogenic and immunomodulatory factors [10,45,48]. Spheroid culture may also preserve osteogenic competence and modify extracellular-vesicle-mediated paracrine signaling [11,13,49]. Together, these properties may improve MSC survival, persistence, and regenerative potential after transplantation. The green and orange panels represent conventional 2D culture and 3D spheroid culture, respectively; the downward arrows indicate the biological consequences associated with each culture strategy.

These changes can influence MSC metabolism, stress responses, secretory activity, migration, immunomodulatory function, and differentiation competence. However, the magnitude and direction of these effects depend on cell source, spheroid size, culture conditions, and manufacturing method and should therefore not be regarded as intrinsic advantages of three-dimensional aggregation in every experimental setting. The principal differences between conventional 2D-expanded MSCs and MSC spheroids are summarized in Figure 2.

### 4.2. Enhanced Survival Under Ischemic Conditions

Spheroid formation can improve resistance to ischemia-like stress. Regmi et al. [47] linked increased viability in 3D MSC aggregates to autophagy and reduced ROS, while Murphy et al. [45] reported lower apoptosis under serum-deprived hypoxic conditions than in dissociated MSCs. These findings support improved stress tolerance in selected spheroid systems.

Hypoxic preconditioning may further modify this response: Ho et al. [15] reported improved survival and bone repair by preconditioned spheroids in a segmental femoral-defect model. ONFH studies using non-spheroid MSCs likewise support the relevance of hypoxic adaptation [50,51], but they provide mechanistic rather than direct evidence for spheroid efficacy.

### 4.3. Enhanced Angiogenic and Paracrine Activity

MSC aggregation can increase proangiogenic paracrine activity. Under ischemia-like conditions, Murphy et al. [45] observed markedly increased VEGF secretion, while Bhang et al. [52] reported greater graft survival, vascular density, perfusion, and VEGF/FGF-2 production after spheroid transplantation in hindlimb ischemia. Other studies have identified changes in angiogenic miRNAs and endothelial responses [48,53].

These findings suggest that spheroids may act as transient paracrine units rather than relying on durable engraftment. However, most evidence is non-ONFH. Hybrid MSC/endothelial spheroids and ONFH co-transplantation studies support the broader concept of coordinated vascular and skeletal regeneration [38,54], but do not establish the superiority of MSC spheroids in the femoral head.

### 4.4. Osteogenic Potential and Bone Regeneration

Spheroid culture can also preserve or enhance osteogenic properties in selected systems. MSC spheroids have shown increased osteogenic differentiation and bone formation in calvarial and femoral-defect models [14,55,56], and endogenous collagen-α2β1-integrin interactions can help maintain an osteogenic phenotype after withdrawal of soluble induction cues [11].

These findings are relevant to ONFH because viable, mechanically competent bone must replace necrotic trabeculae. Nevertheless, osteogenic responses vary with MSC source, passage, spheroid size, preconditioning, biomaterial environment, implantation site, and dose. Three-dimensional morphology alone should, therefore, not be used as a surrogate for osteogenic potency.

### 4.5. Relevance of MSC Spheroids to ONFH

Direct head-to-head evidence demonstrating the superiority of MSC spheroids over equivalent doses of conventional MSC suspensions specifically in ONFH remains limited. Most available evidence originates from bone-defect, ischemic-tissue, inflammatory, and regenerative-angiogenesis models. Accordingly, the literature should be interpreted according to disease specificity: studies of MSC spheroids performed outside ONFH provide indirect mechanistic or proof-of-concept evidence, whereas ONFH studies using non-spheroid MSC interventions provide disease-specific support for particular mechanisms but do not directly establish spheroid efficacy.

The biological properties most relevant to ONFH include improved resistance to environmental stress, enhanced proangiogenic paracrine activity, the preservation of osteogenic competence, and the modulation of inflammatory signaling [10,15,47,48,52,55,56]. Representative preclinical studies and their quantitative outcomes are summarized in Table 2. Quantitative findings from representative non-ONFH studies indicate that some of these effects can be substantial. Under serum-deprived and hypoxic conditions, MSC spheroids secreted up to approximately 100-fold more VEGF than equivalent numbers of dissociated MSCs while exhibiting reduced apoptosis and maintaining comparable osteogenic potential [45]. In a murine hindlimb-ischemia model, spheroid transplantation increased vWF-positive microvessel density from 31.7 ± 14.6 to 99.0 ± 23.6 vessels/mm^2^ and α-SMA-positive mature vessels from 3.0 ± 2.0 to 11.7 ± 4.9 vessels/mm^2^ compared with dissociated-cell transplantation [52]. These findings provide quantitative support for enhanced proangiogenic activity in specific experimental systems.

Importantly, ONFH-specific studies using non-spheroid MSC interventions independently support the disease relevance of several mechanisms proposed to underlie spheroid activity. HIF-1α-overexpressing BM-MSCs improved bone repair and preservation of the femoral head in experimental ONFH [51], while the combined transplantation of MSCs and endothelial progenitor cells enhanced both vascularization and bone regeneration [38]. However, because these studies did not directly compare spheroids with equivalent conventional MSC preparations, they should be regarded as disease-specific mechanistic validation rather than direct evidence of spheroid superiority.

Biological plausibility should not be equated with demonstrated therapeutic efficacy. Improvements in cell survival, trophic-factor secretion, vascular density, or bone formation observed in non-ONFH models do not necessarily predict restoration of subchondral mechanical competence or prevention of femoral-head collapse. Accordingly, the available evidence supports a mechanistic rationale and preclinical proof of concept but does not yet establish that MSC spheroids provide clinically meaningful superiority in ONFH. Dedicated disease-specific studies are required to determine whether the biological advantages observed in other regenerative models translate into durable revascularization, trabecular reconstruction, biomechanical preservation, and prevention of structural collapse.

### 4.6. Limitations of Extrapolating Evidence from Non-ONFH Models to Femoral-Head Osteonecrosis

A major limitation of the current evidence base is that most studies demonstrating advantages of MSC spheroids were not performed in ONFH. Calvarial defects, segmental long-bone defects, hindlimb ischemia, inflammatory models, and general angiogenesis assays reproduce selected components of ONFH pathophysiology but do not reproduce the disease as a whole. Calvarial defects, for example, do not experience the repetitive load-bearing forces present in the femoral head, whereas segmental long-bone defects are generally mechanically stabilized. Hindlimb-ischemia models reproduce tissue hypoperfusion but lack necrotic trabecular architecture, subchondral remodeling, and the risk of femoral-head collapse. Thus, efficacy in these models should be interpreted as support for specific biological mechanisms rather than as direct evidence of disease-modifying efficacy in ONFH.

Reproducibility is further limited by substantial heterogeneity in MSC source, donor characteristics, passage number, spheroid diameter, number of cells per spheroid, aggregation method, culture duration, oxygen conditions, biomaterial carrier, administered dose, and experimental endpoint. Many studies are small proof-of-concept investigations performed within a single laboratory and use short follow-up periods. Consequently, an intervention described generically as an “MSC spheroid” may represent biologically different products across studies. The absence of standardized manufacturing and potency criteria makes quantitative comparison and independent replication difficult.

The available literature is also likely to be affected by the selective reporting of positive proof-of-concept findings. A formal assessment of publication bias is not currently possible because the spheroid literature is small, heterogeneous, and includes diverse experimental models and endpoints. Nevertheless, preferential publication of studies showing improvements in angiogenesis, cell survival, or bone formation could overestimate the apparent consistency of therapeutic benefit. This concern is relevant because the broader clinical MSC literature in ONFH includes neutral findings: BMAC provided no significant additional benefit over core decompression in the randomized study of Pepke et al. [30], whereas Yoon et al. [31] observed increased vascularity without a significant improvement in lesion size or major functional outcomes.

Several factors may therefore contribute to the preclinical-to-clinical translation gap: differences between experimental and human disease severity; variability in MSC products; the loss of potency during scale-up or cryopreservation; inadequate retention or delivery; large or mechanically unstable lesions; insufficient duration of follow-up; and reliance on surrogate endpoints that do not directly measure prevention of collapse or THA. Future studies should prioritize standardized spheroid products, direct comparisons with equal-dose conventional MSCs, clinically relevant ONFH models, biomechanical endpoints, and sufficiently long observation periods.

### 4.7. Combining MSC Spheroids with Biomaterial Delivery Systems

Biomaterials can improve the local retention of MSC spheroids, conform to irregular lesions, and provide matrix-like cues. Fibrin and RGD-modified alginate systems have improved spheroid survival, proangiogenic activity, or mineralization in experimental models [45,57], and hypoxia-preconditioned spheroids in RGD-alginate improved segmental bone repair [15].

For ONFH, injectable spheroid-loaded hydrogels could be delivered through the CD tract to localize cells and prolong local paracrine activity. However, the contributions of spheroid organization, biomaterial signaling, and any added growth factors should be separated experimentally, and efficacy demonstrated in non-ONFH defect models should not be assumed to predict femoral-head preservation.

### 4.8. Spheroid Size and Manufacturing Considerations

Spheroid size is a critical product variable because it determines oxygen and nutrient diffusion and thereby affects metabolism, viability, senescence, secretory activity, and ECM organization. Murphy et al. [12] demonstrated size-dependent oxygen gradients without the obligatory development of a completely anoxic core, while metabolic activity declined as aggregate size increased.

Rovere et al. [49] reported size-dependent senescence, soluble-factor and EV composition, and angiogenic activity, with smaller spheroids showing lower senescence and stronger proangiogenic effects. Yuan et al. [58] similarly identified an intermediate size range that balanced physiological hypoxia with viability and angiogenic activity, underscoring that larger aggregates are not inherently more potent.

Clinical translation therefore requires control of cell number per spheroid, size distribution, morphology, viability, passage, culture conditions, and biological potency. For ONFH, the preferred product is likely to be a reproducible and injectable aggregate that balances survival, adaptive signaling, angiogenic and osteogenic competence, and distribution throughout the lesion.

## 5. Mechanisms Potentially Underlying MSC-Spheroid Activity in ONFH

MSC-spheroid activity is likely to reflect interacting changes in cell–cell/ECM organization, oxygen and nutrient gradients, stress adaptation, and secretory phenotype. The following mechanisms are supported to varying degrees by spheroid studies and ONFH-specific evidence and should be interpreted according to the underlying model.

### 5.1. Hypoxic Adaptation and HIF-1α Signaling: Therapeutic Window and Limitations

A major consequence of MSC aggregation is the establishment of an oxygen microenvironment that differs substantially from conventional monolayer culture. Direct measurements have demonstrated size-dependent changes in oxygen availability and cellular metabolism within MSC spheroids, although appropriately sized aggregates do not necessarily develop a completely anoxic core [12]. Thus, the biological effects of three-dimensional aggregation are more appropriately interpreted as responses to spatial oxygen gradients and hypoxia-associated metabolic adaptation rather than to severe central hypoxia.

HIF-1α is a central regulator of cellular adaptation to reduced oxygen availability and controls metabolic reprogramming, cell survival, and angiogenic signaling, including induction of VEGF. The magnitude of HIF-1α activation within MSC spheroids is influenced by aggregate geometry. Rovere et al. [49] reported higher HIF-1α expression in larger spheroids than in smaller spheroids and conventional 2D cultures, whereas Yuan et al. [58] demonstrated that an intermediate degree of size-dependent physiological hypoxia could provide a favorable balance between cell viability and proangiogenic activity. Hypoxic preconditioning before spheroid formation has likewise been associated with increased VEGF secretion, greater resistance to apoptosis, and improved bone regeneration in experimental models [15].

The relevance of hypoxia-responsive signaling to ONFH is supported by disease-specific studies using non-spheroid MSC preparations. Fan et al. [59] showed that culture of BM-MSCs from rabbits with osteonecrosis under 2% O_2_ increased growth-factor secretion, enhanced cell viability, and reduced apoptosis. Following implantation after core decompression, hypoxia-preconditioned BM-MSCs promoted angiogenesis and osteogenesis more effectively than conventionally cultured cells. Similarly, transplantation of HIF-1α-overexpressing BM-MSCs reduced osteonecrotic areas and enhanced bone repair in glucocorticoid-induced ONFH [51]. However, because these studies did not directly evaluate MSC spheroids, they provide disease-specific mechanistic support for hypoxic adaptation rather than direct evidence of spheroid efficacy in ONFH.

Importantly, hypoxia should not be considered uniformly beneficial. Its biological effects depend on oxygen concentration, duration of exposure, spheroid size, nutrient availability, cell source, and metabolic state. Moderate and transient hypoxia may activate adaptive HIF-dependent signaling, whereas severe or prolonged oxygen deprivation may exceed the adaptive capacity of MSCs, leading to impaired energy metabolism, mitochondrial dysfunction, oxidative stress, reduced proliferation and viability, and altered differentiation. These effects are particularly relevant to ONFH, where the preservation of osteogenic competence is required in addition to proangiogenic activity.

Three-dimensional geometry introduces an additional constraint because increasing aggregate size prolongs diffusion distances for oxygen and nutrients. Large spheroids may therefore develop steep radial gradients in oxygen, glucose, metabolites, and pH. Although a completely anoxic or necrotic core is not inevitable [12], excessive aggregate dimensions can generate metabolically compromised or non-viable central regions. Moreover, Rovere et al. [49] demonstrated size-dependent differences in cellular senescence and angiogenic activity, with smaller spheroids showing lower senescence and stronger proangiogenic effects. These observations indicate that greater intraspheroidal hypoxia should not be assumed to confer greater therapeutic potency.

Thus, spheroid-associated hypoxia should be regarded as a dose- and context-dependent biological stimulus rather than an intrinsic advantage of three-dimensional aggregation. Development of a clinically reproducible MSC spheroid product will require definition of a therapeutic window in which adaptive HIF-dependent signaling is activated without inducing excessive metabolic stress, senescence, loss of viability, or impairment of osteogenic function. Oxygen tension, exposure duration, spheroid size, and nutrient availability should therefore be considered interconnected determinants of product potency.

### 5.2. Enhanced Cell Survival, Autophagy and Resistance to Apoptosis

Spheroid formation preserves cell contacts and endogenous ECM that are disrupted during enzymatic detachment and can thereby improve resistance to transplantation-associated stress. Increased BCL-2/BAX ratios and trophic-factor expression have been reported in spheroids [60], and several studies show improved survival under ischemia-like conditions [45,52].

Autophagy appears to contribute to this response: Regmi et al. [47] demonstrated increased autophagic activity, reduced ROS, and reduced apoptosis in 3D MSC spheroids, with a loss of the survival advantage after autophagy inhibition. In ONFH, such early stress resistance may prolong paracrine activity, but an excessive aggregate size can shift adaptation toward senescence or cell death.

### 5.3. Enhancement of the Proangiogenic Secretome

Spheroid culture can increase the secretion of proangiogenic mediators. Human MSC spheroids under ischemia-like conditions showed increased VEGF [45], and cord-blood MSC spheroids improved VEGF/FGF-2 production, microvascular density, and perfusion in hindlimb ischemia [52]. These effects likely reflect coordinated trophic signaling rather than VEGF alone.

Because revascularization is central to ONFH repair, this phenotype is mechanistically relevant, and ONFH studies combining MSCs with endothelial progenitor cells support simultaneous targeting of vascular and skeletal compartments [38]. However, direct evidence that spheroid-specific secretome changes improve femoral-head preservation remains limited.

### 5.4. Preservation of Osteogenic Activity

Three-dimensional aggregation can preserve osteogenic signaling through endogenous ECM and cell adhesion. Osteogenically preconditioned MSC spheroids maintained their phenotype after the withdrawal of soluble cues through collagen-α2β1-integrin interactions [11], supporting a role for self-generated matrix in sustaining osteogenic competence.

This property is relevant to the replacement of necrotic trabeculae, but osteogenic responses vary with source, passage, size, differentiation status, culture conditions, and biomaterial environment. Spheroid formation should therefore not be assumed to enhance osteogenesis in every setting.

### 5.5. Angiogenesis-Osteogenesis Coupling

ONFH repair requires coordinated vascular and skeletal regeneration: vessels provide oxygen, nutrients, and progenitor access, while viable bone reconstruction is required for mechanical recovery. ONFH studies of hypoxia-preconditioned or HIF-1α-modified MSCs and combined MSC/endothelial progenitor-cell delivery support the therapeutic relevance of targeting both processes [38,51,59].

MSC spheroids may contribute to this coupling by combining proangiogenic paracrine activity with preserved osteogenic competence. This remains a mechanistic hypothesis rather than a demonstrated ONFH-specific advantage, and the complete causal sequence has not been validated in direct spheroid-versus-2D comparisons.

### 5.6. Immunomodulatory Activity

Spheroid formation can alter MSC immunomodulatory activity. Bartosh et al. [10] reported increased TSG-6 and other cytoprotective mediators and greater anti-inflammatory activity after aggregation. Such effects could reduce secondary inflammatory injury in ONFH, but direct evidence identifying TSG-6 or related mediators as major effectors of spheroid therapy in ONFH is lacking. This mechanism should therefore be regarded as biologically plausible rather than disease-specific.

### 5.7. Extracellular Vesicle-Mediated Signaling

MSC therapeutic activity is partly mediated by extracellular vesicles (EVs), and three-dimensional culture can alter EV production and cargo. Spheroid size influences senescence, soluble secretome composition, EV release, and angiogenic potency [49].

ONFH-specific evidence supports the broader relevance of EV signaling: exosomes from hypoxia-preconditioned BM-MSCs increased vascular parameters and reduced bone loss in rat steroid-induced ONFH [61], while exosomes from iPSC-derived MSCs promoted angiogenesis through PI3K/AKT signaling and reduced bone loss [62].

A meta-analysis of 12 preclinical studies reported improvements in bone mineral density, trabecular architecture, and vascular parameters after stem-cell-derived exosome therapy [63]. However, all included studies were performed in rats, and the contribution of EVs specifically to MSC-spheroid therapy remains to be established.

### 5.8. Integrated Mechanism of MSC-Spheroid-Mediated Repair in ONFH

**Figure 3:**
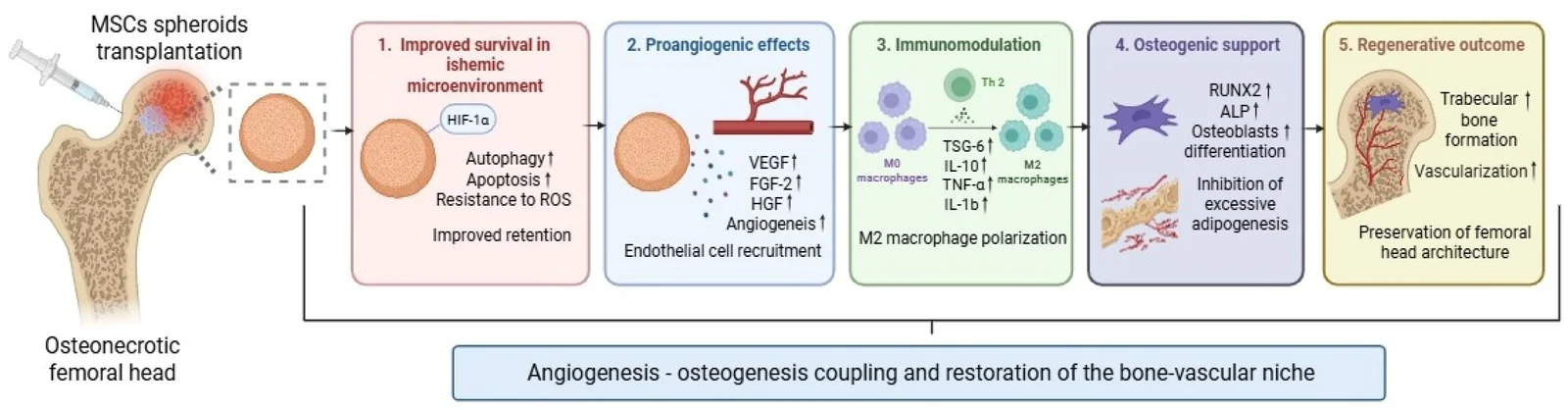
Proposed mechanisms by which MSC spheroids may promote angiogenesis–osteogenesis coupling in ONFH. The schematic illustrates the major cellular and molecular mechanisms through which MSC spheroids may improve regeneration within the ischemic and structurally compromised femoral head. Following local transplantation (left), MSC spheroids encounter a hypoxic, nutrient-deprived, inflammatory and oxidative microenvironment. (1) Improved survival in the ischemic microenvironment: three-dimensional cell–cell and cell–ECM interactions, together with adaptive hypoxic signaling, are associated with HIF-1α activation, enhanced autophagy, reduced apoptosis and oxidative stress, and improved cell persistence [12,15,45,47,49,58]. These responses may increase the resistance of transplanted MSCs to the hostile ONFH microenvironment. (2) Proangiogenic effects: MSC spheroids exhibit enhanced secretion of angiogenic mediators, particularly VEGF and FGF-2, with additional contributions from other paracrine factors such as hepatocyte growth factor (HGF) [45,52]. These mediators promote endothelial-cell recruitment, survival, migration, and neovascularization, potentially facilitating the restoration of local microvascular perfusion. (3) Immunomodulation: spheroid formation enhances the immunoregulatory phenotype of MSCs, including increased expression of tumor necrosis factor-stimulated gene-6 (TSG-6) and other anti-inflammatory mediators [10]. These signals may suppress pro-inflammatory pathways and favor the resolution of inflammation. However, specific effects on IL-10, TNF-α/IL-1β, and M2 macrophage polarization shown in the schematic should be interpreted as mechanistically supportive rather than uniformly demonstrated in ONFH-specific MSC-spheroid studies. (4) Osteogenic support: spheroid culture can preserve or enhance osteogenic competence through endogenous extracellular matrix–integrin signaling, including collagen–α2β1-integrin interactions, and it is associated with increased osteogenic differentiation and mineralization in experimental bone-regeneration models [11,14,15]. RUNX2 and ALP shown in the panel represent key markers of osteogenic differentiation. (5) Regenerative outcome: the combined pro-survival, proangiogenic, immunomodulatory, and osteogenic effects are proposed to improve vascularization and trabecular bone regeneration and thereby support preservation of femoral-head architecture. Importantly, the prevention of femoral-head collapse by MSC spheroids has not yet been directly demonstrated in controlled ONFH studies. This final step therefore represents a proposed translational outcome rather than an established therapeutic effect. The black arrows indicate the proposed sequence of biological effects; ↑ indicate increased responses, respectively. Most supporting evidence derives from non-ONFH experimental models.

Taken together, the current evidence suggests that MSC spheroids may act through an integrated network in which improved stress adaptation and cell survival sustain paracrine, angiogenic, immunomodulatory, and osteogenic functions. These interconnected mechanisms and their proposed contribution to restoration of the bone–vascular niche in ONFH are summarized in Figure 3.

However, no single study has demonstrated the complete sequence from spheroid implantation through molecular adaptation, revascularization, trabecular reconstruction, and the prevention of femoral-head collapse. The proposed pathway should therefore be regarded as an evidence-based integrative model rather than a fully validated ONFH-specific mechanism.

## 6. Strategies to Enhance the Therapeutic Potential of MSC Spheroids for ONFH

Spheroid formation alone is unlikely to overcome all features of the ONFH microenvironment. The strategies discussed below aim to improve cell survival and delivery, vascularization, bone regeneration, and mechanical support while balancing potential biological benefits against increasing manufacturing and regulatory complexity. The principal strategies for enhancing the therapeutic potential of MSC spheroids in ONFH, together with their biological rationale, supporting evidence, potential advantages, and major limitations, are summarized in Table 3.

### 6.1. Hypoxic Preconditioning of MSC Spheroids

Given the dose-dependent role of hypoxic adaptation described in Section 5.1, controlled preconditioning before spheroid formation represents a rational strategy for improving resistance to the ischemic ONFH microenvironment. Ho et al. [15] reported that MSCs exposed to 1% O_2_ before spheroid formation showed reduced apoptosis and increased VEGF secretion while preserving osteogenic competence; subsequent implantation improved repair of critical-sized femoral defects compared with preconditioned individual MSCs. However, this evidence derives from a non-ONFH model.

The translational objective should therefore not be to maximize hypoxic signaling but to identify an exposure regimen that preserves viability and osteogenic competence while inducing a reproducible adaptive phenotype. Oxygen concentration, the duration of exposure, spheroid size, recovery period, and post-preconditioning potency should be optimized together. Excessive or prolonged hypoxia may reduce proliferation, alter differentiation, increase metabolic stress and senescence, and contribute to non-viable central regions in larger aggregates.

### 6.2. Optimization of Spheroid Size and Cellular Organization

Spheroid size affects oxygen/nutrient diffusion, metabolism, apoptosis, proliferation, and secretory activity [45,58]. Moderate physiological hypoxia can support angiogenic activity, whereas larger aggregates may develop unfavorable gradients, central dysfunction, and non-viable regions. Thus, size should be optimized for function rather than maximized.

For ONFH, standardized small-to-intermediate spheroids may also distribute more uniformly through the necrotic region than a limited number of large aggregates. Tissue source, passage, aggregation time, culture medium, oxygen conditions, and formation method should be controlled as product-critical variables.

### 6.3. Biomaterial-Assisted Delivery of MSC Spheroids

Injectable or implantable biomaterials can improve spheroid retention, provide ECM-like cues, and influence local mechanics. Fibrin and RGD-alginate systems have improved survival, VEGF secretion, or mineralization in spheroid models [45,57], while hydrogel viscoelasticity can regulate osteogenic activity [64].

For ONFH, injectable hydrogels are attractive because they can be delivered through the CD tract and conform to irregular lesions. Their composition, degradation, mechanics, and contribution to efficacy must nevertheless be optimized independently of the cellular component.

### 6.4. Prolongation of the MSC-Spheroid Secretome

Secretome-sequestering biomaterials may prolong local paracrine activity without requiring permanent donor-cell engraftment. Sulfated alginate can retain heparin-binding factors released by MSC spheroids while preserving endothelial bioactivity [72]. For ONFH, such systems could extend local presentation of trophic mediators during the early post-implantation period, although the added benefit over simpler carriers remains to be demonstrated.

### 6.5. Oxygen-Generating and Microenvironment-Modulating Biomaterials

Temporary oxygen delivery may protect transplanted cells during severe early ischemia. In rabbit ONFH, CaO_2_/gelatin microspheres within a tissue-engineered scaffold sustained oxygen release, reduced grafted-cell apoptosis, and improved angiogenic and osteogenic outcomes [39]. Although conventional BM-MSCs rather than spheroids were used, the principle may be applicable to spheroid delivery.

Oxygen release requires careful control because excessive oxygen can increase ROS and suppress adaptive hypoxic signaling. Biomaterials may also target inflammation. For example, an injectable hydrogel modulating immune infiltration improved the ONFH microenvironment in a recent preclinical study [65].

### 6.6. Controlled Delivery of Angiogenic and Osteogenic Factors

The controlled delivery of bioactive factors could complement spheroid-based repair. FGF-2 is particularly relevant because local gelatin-hydrogel delivery has progressed to clinical evaluation in precollapse ONFH, with reported bone regeneration and joint preservation [66,67,68].

Combining FGF-2 with MSC spheroids is therefore biologically plausible but has not been clinically validated. VEGF or FGF-2 may be useful for early vascular support, whereas BMP signaling could enhance later bone formation; temporally staged delivery may therefore be preferable to simultaneous exposure.

ONFH-specific preclinical work with sustained rhBMP9 delivery further supports biomaterial-mediated coordination of osteogenic and angiogenic signaling [73]. Future combinations should define dose, release kinetics, and the independent contribution of each component.

### 6.7. Prevascularization and Msc–Endothelial-Cell Co-Spheroids

Including endothelial cells or progenitors may accelerate vascularization. In rabbit ONFH, the co-transplantation of MSCs and endothelial cells improved angiogenesis and bone regeneration [69], while spatially organized MSC-spheroid grafts enhanced neovascularization in a non-ONFH ischemia model [70].

Heterotypic MSC–endothelial spheroids are therefore conceptually attractive, but an additional cell type increases manufacturing, safety, and regulatory complexity. Their value in ONFH should be established against simpler single-cell-type products.

### 6.8. Biomechanical Considerations and the Need for Structural Support

An important limitation of purely cellular or hydrogel-based therapies is their inability to provide immediate restoration of the mechanical integrity of a structurally weakened femoral head. This issue becomes increasingly important as the necrotic lesion enlarges, involves the principal load-bearing region, or approaches subchondral collapse. Although regenerative therapies may stimulate vascular and bone formation, newly deposited tissue requires time to mature, mineralize, and establish sufficient trabecular connectivity to tolerate physiological loading. During this interval, continued joint loading may perpetuate microfracture and structural deformation despite biologically favorable repair.

The biomechanical challenge in ONFH is therefore substantially different from that in several experimental models commonly used to evaluate MSC spheroids. Calvarial defects do not reproduce repetitive compressive and shear loading of the femoral head, whereas segmental long-bone defects are generally mechanically stabilized. In ONFH, regeneration must occur within a weight-bearing osteochondral structure in which the preservation of the subchondral plate and trabecular architecture is essential for maintaining femoral-head sphericity. Successful bone formation in non-load-bearing models therefore cannot be assumed to predict prevention of collapse.

Cell therapy is most likely to be effective when sufficient native architecture remains to provide temporary mechanical support. Small precollapse lesions may therefore be amenable to core decompression combined with locally delivered MSCs or MSC spheroids. In contrast, larger lesions with substantial loss of trabecular support may require additional osteoconductive or mechanically reinforcing components. Thus, patient and treatment selection should be based not only on biological disease stage but also on lesion size, location, and residual mechanical competence.

The study of Guo et al. [71], in which a bioactive magnesium scaffold was combined with BM-MSC-loaded three-dimensional microspheres in rabbit ONFH, illustrates this principle. The scaffold provided structural support while the cellular component promoted a regenerative osteogenic–angiogenic microenvironment. Although these microspheres are not equivalent to scaffold-free MSC spheroids, the study suggests that biological and mechanical functions may need to be addressed simultaneously. Future constructs should therefore be assessed not only for histological bone formation and vascularization but also for trabecular architecture, load transfer, compressive competence, fatigue behavior, scaffold degradation, the preservation of femoral-head geometry and resistance to collapse.

### 6.9. Toward an Integrated Therapeutic Platform for ONFH

No single enhancement strategy addresses all pathological components of ONFH. A pragmatic early-stage platform could combine CD, standardized spheroids, and an injectable ECM-mimicking hydrogel; more complex additions such as growth factors, oxygen-generating materials, endothelial cells, or structural scaffolds should be introduced only when they provide a demonstrable incremental benefit.

Increasing technological complexity adds a manufacturing, cost, safety, and regulatory burden. Development should therefore prioritize reproducible interventions with favorable benefit-to-complexity ratios and tailor biological versus mechanically supportive components to lesion stage and structural requirements.

## 7. Translational Challenges and Roadmap to Clinical Application

Despite their promising biological properties, the translation of MSC spheroids from experimental models to clinical application in ONFH requires the resolution of several interrelated manufacturing, regulatory, safety, and commercial challenges. A clinically viable spheroid therapy must be developed as a well-defined and reproducible cell product rather than simply as a three-dimensional culture format. This requires scalable manufacturing, validated critical quality attributes and potency assays, preservation of function during storage and transport, comprehensive product-specific safety assessment, and a regulatory strategy appropriate to the intended route of administration and product composition. In addition, increasing product complexity through incorporation of biomaterials, growth factors, or additional cell types must provide sufficient therapeutic benefit to justify the associated manufacturing, regulatory, and economic burden. The following sections outline the principal translational challenges and key considerations for advancing MSC spheroid-based therapies for ONFH toward clinical evaluation.

### 7.1. Manufacturing Reproducibility and Scalability

The clinical translation of MSC spheroids requires control of both conventional MSC variability and aggregate-specific manufacturing variability. Donor characteristics, tissue source, passage number, population-doubling history, culture medium, raw materials, oxygen conditions, and expansion platform can alter MSC phenotype. Spheroid formation introduces additional variables, including cell number per aggregate, diameter and size distribution, aggregation time, compaction, endogenous ECM deposition, oxygen/nutrient gradients, shear stress, and harvesting procedure. Consequently, two products described simply as “MSC spheroids” may differ substantially in viability, secretory activity, stress resistance, and differentiation competence.

Manufacturing should therefore be based on predefined critical quality attributes and critical process parameters. Relevant quality attributes may include cell identity, viability, dose, spheroid diameter and size distribution, morphology, purity, sterility, and mechanism-related potency. Critical process parameters include passage number, inoculation density, aggregation platform, culture duration, oxygen tension, medium composition, agitation, and harvesting conditions. Laboratory-scale hanging-drop or static low-attachment methods may not be suitable for manufacturing the large numbers of uniform aggregates required clinically; automated microwells, closed systems, and controlled suspension or bioreactor platforms may offer more scalable alternatives [74,75,76].

### 7.2. Cryopreservation, Storage and Post-Thaw Function

Cryopreservation is essential for a practical allogeneic “off-the-shelf” product but represents a potentially important source of functional variability. Post-thaw viability alone is unlikely to be an adequate indicator of product equivalence because freeze–thaw procedures can influence recovery, membrane integrity, metabolic activity, paracrine function, immunomodulatory potency, and differentiation capacity [75]. These considerations may be particularly important for spheroids because cryoprotectant penetration and thermal transfer may differ between peripheral and central regions of an aggregate.

A key development question is whether the final product should be cryopreserved as intact spheroids or whether expanded MSCs should be cryopreserved first and aggregated after thawing. Product-specific studies should evaluate post-thaw morphology, viability, aggregate integrity, recovery, mechanism-related potency, storage duration, transport conditions, thawing procedure, and permissible post-thaw holding time. Cryopreservation should therefore be considered part of product development rather than a purely logistical step.

### 7.3. Potency, Release Criteria, Quality Control and Regulatory Pathways

Conventional MSC identity markers are insufficient to establish therapeutic potency. For an ONFH-directed spheroid product, potency testing should be linked to the proposed mechanism of action and should ideally combine complementary functional readouts. Candidate assays include survival under ischemia-like conditions, the secretion of defined trophic mediators, endothelial-cell migration or angiogenic activity, the preservation of osteogenic competence, and, where relevant, immunomodulatory function. Because lengthy functional assays may be impractical for routine batch release, rapid validated surrogate assays that correlate with more comprehensive functional testing will ultimately be required [77].

Final release specifications should be predefined and justified and may include identity, cell number or dose, viability, purity, spheroid diameter and size distribution, morphology, sterility, mycoplasma, endotoxin, and mechanism-related potency. Combination products incorporating hydrogels, scaffolds, growth factors, or additional cell types will require additional specifications for individual components and for the final combined product. Manufacturing changes introduced during scale-up should be accompanied by comparability studies demonstrating the preservation of critical biological properties.

Regulatory classification will depend on jurisdiction, manufacturing process, intended use, and product composition. In the European Union, substantially manipulated stem-cell products or cells used for a different essential function generally fall within the Advanced Therapy Medicinal Product framework; incorporation of a medical-device component may lead to consideration as a combined ATMP. In the United States, the applicable pathway depends on whether the product satisfies the criteria for regulation solely as an HCT/P; substantially manipulated or non-homologously used products generally require premarket evaluation within the biological-product framework. Early interaction with regulatory authorities will therefore be important because classification influences manufacturing, nonclinical testing, clinical-trial design, and marketing authorization requirements.

### 7.4. Safety Considerations and Risk Assessment

The safety profile of MSC spheroids should not be assumed to be identical to that of dissociated MSC preparations. Three-dimensional aggregation changes physical dimensions, cell–cell and cell–ECM interactions, metabolic state, secretory activity, and potentially post-transplantation persistence. Product-specific assessment should therefore address local and systemic safety rather than relying solely on clinical experience with conventional MSC suspensions.

Potential concerns include ectopic or inappropriate tissue formation, particularly when spheroids are combined with osteoinductive factors or biomaterials. Long-term studies should assess whether newly formed mineralized or fibrotic tissue remains restricted to the intended lesion. Biodistribution and persistence should also be characterized because prolonged local retention may extend therapeutic activity but may also prolong exposure to angiogenic, immunomodulatory, or differentiation signals.

Route-dependent vascular safety requires particular attention. MSC preparations may exhibit procoagulant activity that varies with tissue source, dose, passage, and manufacturing conditions [78]. Intact spheroids are substantially larger than dissociated cells; consequently, intravascular administration of intact aggregates would raise an additional theoretical concern regarding microvascular obstruction. Local implantation through a core-decompression tract may reduce systemic exposure, although inadvertent vascular entry and local accumulation should still be considered.

MSC immunomodulatory activity should not be interpreted as complete immunological invisibility. Allogeneic products may interact with innate and adaptive immune systems, while residual serum proteins, cryoprotectants, antibiotics, or other process-related impurities may contribute to inflammatory or hypersensitivity reactions. Product development should therefore include appropriate immunogenicity and inflammatory-response monitoring.

Tumor-related safety requires distinction between malignant transformation of the administered product and potential effects of MSC-derived signals on pre-existing malignant cells. Meta-analyses of conventional MSC trials have not demonstrated an increased incidence of malignancy attributable to MSC administration, although follow-up and adverse-event reporting remain heterogeneous [79]. At the same time, experimental literature indicates that MSCs can support tumor-associated angiogenesis, immune evasion, or stromal remodeling under some conditions [80]. These observations do not demonstrate that MSC-spheroid therapy causes cancer, but they justify control of genomic stability, abnormal growth, extensive ex vivo expansion, careful patient selection, and appropriate long-term surveillance.

### 7.5. Commercialization and Clinical Development

Commercial translation will depend not only on biological efficacy but also on whether MSC spheroids can be manufactured, stored, released, and delivered at a cost compatible with orthopedic practice. Major cost drivers include donor qualification, cell banking, GMP expansion, spheroid formation, clean or closed-system operation, quality-control and potency testing, cryopreservation, transport, and the surgical delivery procedure. Addition of recombinant factors, endothelial cells, genetically modified cells, or complex scaffolds would further increase cost and regulatory burden.

Autologous and allogeneic approaches involve different commercial trade-offs. Autologous products avoid donor–recipient mismatch but require patient-specific manufacturing and individualized release testing. Allogeneic banked products offer greater potential for standardization and scale but require robust donor qualification, cell-bank characterization, cryopreservation, batch consistency, and confirmation that prolonged expansion does not compromise potency.

Formal cost-effectiveness data for MSC-spheroid therapy in ONFH are currently unavailable. Future trials should therefore incorporate health-economic outcomes together with biological and clinical endpoints, including prevention or delay of femoral-head collapse, avoidance of THA, quality-adjusted survival, revision procedures, and long-term healthcare utilization. A biological advantage over conventional MSCs will not by itself justify clinical adoption unless the magnitude and durability of benefit outweigh the additional manufacturing and procedural complexity.

## 8. Conclusions

MSC spheroids are a biologically plausible strategy for addressing several limitations of conventional MSC suspensions, particularly poor retention, stress sensitivity, and the loss of tissue-like cell organization. Preclinical evidence indicates that three-dimensional aggregation can modify survival, paracrine signaling, angiogenic activity, and osteogenic competence, but these effects are product- and model-dependent and most supporting studies are not ONFH-specific.

Therapeutic superiority over equivalent conventional MSC preparations has not been established in ONFH. Future development should therefore prioritize standardized spheroid products, direct disease-specific comparisons, clinically relevant structural and biomechanical endpoints, validated potency and release assays, long-term safety, scalable GMP-compatible manufacturing, and evidence that any added complexity produces durable femoral-head preservation with an acceptable benefit–risk and cost profile.

## Figures and Tables

**Table 1:** Quantitative summary of representative preclinical and clinical studies of MSC-based therapy for ONFH.

Study	Cell Type	Study Design/ONFH Model	Delivery Strategy	Representative Quantitative Outcomes	Main Limitations
**Preclinical studies**					
Chen et al. [33]	hUC-MSCs	Preclinical study; steroid-induced ONFH model	CD + MSC-loaded pluronic F127 hydrogel	At 8 weeks, local delivery of ~1 × 10^6^ hUC-MSCs after CD significantly increased BV/TV, Tb.Th, and Tb.N and decreased Tb.Sp (n = 7/group), with enhanced microvascular regeneration compared with ONFH controls.	Mechanism and efficacy require clinical validation
Chen et al. [34]	Synovial fluid-derived MSCs	Preclinical study; steroid-induced rabbit ONFH model	CD + MSC-loaded alginate beads	At 6 weeks, femoral-head BMD and sphericity were significantly better preserved with CD + SF-MSC-loaded alginate beads than with control treatments (*p* < 0.005), with enhanced bone regeneration.	Short 6-week follow-up; biomaterial contribution cannot be completely separated from MSC effects
Xu et al. [38]	BM-MSCs + endothelial progenitor cells (EPCs)	Preclinical study; steroid-induced rabbit ONFH model	CD + 3D CMC/alginate scaffold containing 5 × 10^6^ BM-MSCs + 5 × 10^6^ EPCs	At 12 weeks, the CMC/ALG scaffold containing 5 × 10^6^ BMSCs + 5 × 10^6^ EPCs produced significantly greater osteogenesis and angiogenesis and lower adipogenesis than CD, CMC/ALG, CMC/ALG/BMSC, and CMC/ALG/EPC treatments.	Preclinical rabbit model; 12-week follow-up; complex combined cell-scaffold intervention makes it difficult to isolate the individual contribution of BMSCs, EPCs, and the scaffold.
Wang et al. [39]	BM-MSCs in oxygen-generating tissue-engineered scaffold	Preclinical study; steroid-induced rabbit ONFH model	CD + BM-MSCs + PCL/nHA scaffold + hydrogel + CaO_2_/gelatin microspheres	Sustained O_2_ release for 19 days; improved BMSC survival under 1% O_2_. In vivo, the oxygen-generating scaffold significantly increased angiogenesis and osteogenesis and reduced local cell apoptosis compared with the scaffold without oxygen-generating microspheres.	Preclinical rabbit model; complex multicomponent scaffold; the individual contributions of oxygen delivery, BMSCs, hydrogel, and PCL/nHA scaffold are difficult to separate.
Guzman et al. [40]	PDGF-BB-overexpressing MSCs	Preclinical study; steroid-induced rabbit ONFH	CD + collagen/alginate hydrogel containing genetically modified MSCs	At 8 weeks, PGK-PDGF-BB-MSC treatment significantly reduced empty lacunae compared with CD, hydrogel, and unmodified MSC treatments (*p* < 0.001), while increasing osteoclast density and angiogenesis (both *p* < 0.01); BMD showed a trend toward improvement versus CD (*p* = 0.074).	Preclinical rabbit model; short-term follow-up; genetically modified MSCs raise additional safety and regulatory concerns; the combined CD–hydrogel–cell intervention complicates attribution of efficacy to PDGF-BB overexpression alone.
**Clinical studies**					
Zhao et al. [29]	Autologous BM-MSCs	Randomized clinical study; 100 patients with early-stage ONFH	CD + local implantation of 2 × 10^6^ cells	At 60 months, femoral-head survival was 96.2% (51/53) versus 77.3% (34/44), and disease progression occurred in 3.8% (2/53) versus 22.7% (10/44) with BM-MSC implantation versus CD alone. THA conversion was 0% versus 11.4%, respectively.	Single-center study; heterogeneous ONFH etiology
Gómez-Barrena et al. [7]	Autologous BM-MSCs	Multicenter ORTHO2 clinical study; early/precollapse ONFH; ≥5-year follow-up	Intraosseous administration after CD; 140 × 10^6^ cells	Femoral-head preservation with bone regeneration: 80% (16/20) at 12 months; no progression or THR: 76.2% (16/21) at 5 years; THR conversion: 19% (4/21).	Non-randomized study; relatively limited cohort; selected early-stage patients
Pepke et al. [30]	Autologous BMAC	Prospective randomized study; 24 patients/25 hips; ARCO II	CD + 10 mL BMAC into necrotic region	At 2 years, HHS was 83 vs. 77 and VAS was 2.2 vs. 3.1 for CD + BMAC versus CD alone, respectively; femoral-head survival without progression or THA was 63.6% (7/11) vs. 57.1% (8/14), with no significant between-group differences.	Small cohort; BMAC is heterogeneous and contains relatively few MSCs; short follow-up
Kang et al. [36]	Autologous BM- MSC	Matched-pair clinical study; 100 patients/106 hips	CD + intraosseous bone marrow cell implantation	THA conversion: 28.3% vs. 49.0% (cell therapy vs. CD alone); greatest benefit observed in ARCO I–II disease.	Retrospective design; treatment did not significantly prevent ARCO stage progression
Yoon et al. [31]	Autologous BM-MSCs	Phase I/IIa clinical study; 18 hips with lesions ≥30%	6-mm drilling + local implantation; 1 × 10^8^ cells	Radiographic failure occurred in 14.3% of ARCO 2 hips and 50% of ARCO 3a hips. THA was performed in 18.2% (4/22) of hips, while the combined necrotic angle decreased by >20° in 27.3% (6/22). No significant changes were observed in VAS or HHS.	No significant reduction in necrotic lesion size or improvement in major functional scores; small uncontrolled cohort
Chen et al. [32]	Allogeneic UC-MSCs	Clinical study; 9 patients/9 hips; ARCO II–IIIa	Intra-arterial infusion	Among 9 treated hips (5 ARCO II and 4 ARCO IIIa), the oxygen delivery index increased at 3 days, and necrotic volume on MRI was significantly reduced at 12 and 24 months after intra-arterial hUC-MSC infusion.	Very small sample (n = 9), retrospective uncontrolled design, heterogeneous ARCO stages, and no comparator group; cell homing and differentiation were not directly demonstrated.

Note: Clinical and preclinical studies are presented separately because of substantial differences in study design, outcome measures, and follow-up duration. Quantitative outcomes are intended for descriptive comparison and should not be interpreted as a formal meta-analysis or as direct evidence of comparative efficacy across studies. Abbreviations: AD-MSCs, adipose-derived mesenchymal stromal cells; BMAC, bone marrow aspirate concentrate; BM-MSCs, bone marrow-derived mesenchymal stromal cells; CD, core decompression; CMC, carboxymethyl chitosan; EPCs, endothelial progenitor cells; hUC-MSCs, human umbilical cord-derived mesenchymal stromal cells; ONFH, osteonecrosis of the femoral head; PCL/nHA, polycaprolactone/nano-hydroxyapatite; PDGF-BB, platelet-derived growth factor-BB; THA, total hip arthroplasty.

**Table 2:** Representative preclinical studies providing direct or indirect mechanistic support for MSC-spheroid-based therapy in ONFH.

Study	MSC Source	Spheroid Strategy	Experimental Model	Representative Quantitative Outcomes	Evidence Level and Relevance to ONFH
Bartosh et al. [10]	Human BM-MSCs	3D aggregation into spheroids	In vitro and in vivo inflammatory models	After 3 days, MSC spheroids showed ~40–61-fold higher TSG-6 and ~34–82-fold higher IL-8 expression than adherent MSCs, with enhanced anti-inflammatory activity.	Indirect mechanistic evidence (non-ONFH inflammatory models); supports immunomodulatory and cytoprotective mechanisms relevant to the inflammatory ONFH microenvironment
Murphy et al. [45]	Human BM-MSCs	Hanging-drop spheroids; 15,000 cells/spheroid selected for subsequent experiments; fibrin hydrogel	Ischemia-like in vitro conditions: serum deprivation + 1% O_2_	VEGF secretion: up to ~100-fold higher than dissociated MSCs; apoptosis reduced; osteogenic potential preserved.	Indirect mechanistic evidence (in vitro ischemia-like conditions); supports enhanced survival, proangiogenic signaling, and preservation of osteogenic competence relevant to ONFH
Bhang et al. [52]	Human cord blood-derived MSCs	Hanging-drop MSC spheroids	Mouse hindlimb ischemia	vWF-positive vessels: 99.0 ± 23.6 vs. 31.7 ± 14.6 vessels/mm^2^; α-SMA-positive mature vessels: 11.7 ± 4.9 vs. 3.0 ± 2.0 vessels/mm^2^.	Indirect mechanistic evidence (hindlimb-ischemia model); supports enhanced angiogenesis and restoration of microvascular perfusion relevant to ONFH
Yamaguchi et al. [14]	Rat BM-MSCs	Spheroids generated in low-binding plates	Rat calvarial bone-defect model	At 4 weeks, MSC spheroids significantly enhanced new-bone formation, osteogenic differentiation, and mineralization versus monolayer MSCs and controls (*p* < 0.05).	Indirect mechanistic evidence (calvarial bone-defect model); supports enhanced osteogenic differentiation and bone regeneration relevant to ONFH
Murphy et al. [11]	Human BM-MSCs	Osteogenically preconditioned MSC spheroids in collagen hydrogel	In vitro osteogenic model	15,000-cell MSC spheroids showed greater ALP activity and calcium deposition than dissociated MSCs; α2β1-integrin blockade reduced osteogenic marker retention (n = 5).	Indirect mechanistic evidence (osteogenic model); relevant to preservation of osteogenic competence in ONFH
Ho et al. [15]	Human BM-MSCs	Hypoxic preconditioning followed by spheroid formation and RGD-alginate hydrogel delivery	Critical-sized segmental femoral defect in rats	Preconditioning at 1% O_2_ for 3 days enhanced VEGF secretion and osteogenic potential of 15,000-cell MSC spheroids, which significantly improved healing of 6-mm femoral defects over 12 weeks (*p* < 0.05).	Indirect mechanistic evidence (critical-sized femoral bone-defect model); supports hypoxic adaptation, angiogenesis–osteogenesis coupling, and bone regeneration relevant to ONFH
Ohori-Morita et al. [56]	Human BM-MSCs	Spheroids generated using neurosphere medium and continuous shaking culture	Critical-sized rat femoral bone defect	At 3 weeks, NM-spheroids significantly increased bone mineral content, bone volume, and BMD in 5 × 3-mm rat femoral defects (n = 4–8; *p* < 0.05), with near-complete defect closure.	Indirect mechanistic evidence (critical-sized femoral bone-defect model); supports improved MSC persistence and bone regeneration relevant to ONFH
Song et al. [54]	Human MSCs + endothelial colony-forming cells (ECFCs)	Hybrid MSC/ECFC spheroids	Murine hindlimb ischemia	At 28 days, hybrid MSC/ECFC spheroids significantly increased blood perfusion and ILB4^+^ capillary and α-SMA^+^ vessel densities in ischemic limbs (n = 5), with <1% dead cells within spheroids.	Indirect mechanistic evidence (hindlimb-ischemia model); supports enhanced vascularization and restoration of microvascular perfusion relevant to ONFH

Note: Most studies provide indirect mechanistic evidence from non-ONFH models. Because of substantial heterogeneity in MSC sources, spheroid-generation methods, experimental models, outcome measures, and follow-up duration, quantitative outcomes should be interpreted descriptively and not as direct evidence of comparative efficacy in ONFH. Abbreviations: BM-MSCs, bone marrow-derived mesenchymal stromal cells; ECM, extracellular matrix; ECFCs, endothelial colony-forming cells; EVs, extracellular vesicles; FGF-2, fibroblast growth factor-2; ONFH, osteonecrosis of the femoral head; RGD, arginine-glycine-aspartic acid; TSG-6, TNF-α-stimulated gene/protein 6; VEGF, vascular endothelial growth factor.

**Table 3:** Strategies for enhancing the therapeutic potential of MSC spheroids in ONFH.

Strategy	Mechanism	Expected Therapeutic Benefit	Representative Evidence	Major Limitations	Translational Readiness
Hypoxic preconditioning	Activation of HIF-1α-dependent cytoprotective and proangiogenic signaling	↑ survival, ↓ apoptosis, ↑ VEGF, enhanced angiogenesis and osteogenesis	Hypoxia-preconditioned MSC spheroids improved vascularization and bone repair [15]	Optimal O_2_ level and exposure time require standardization; excessive hypoxia may impair osteogenesis	Relatively high
Optimization of spheroid size and cellular organization	Control of oxygen/nutrient gradients, metabolism, apoptosis, secretome, and ECM organization	Improved viability, angiogenic potency, injectability, and product reproducibility	Size-dependent effects also demonstrated in MSC-spheroid studies [45]	Optimal size for ONFH remains undefined; manufacturing variability	Relatively high
Injectable hydrogel-assisted delivery	Local retention, protection from mechanical stress, ECM-like signaling, and controlled cell migration	↑ retention and survival, prolonged paracrine activity, enhanced angiogenesis and osteogenesis	Fibrin and RGD-alginate systems improved MSC-spheroid survival, VEGF secretion, and bone formation [45,57,64]	Hydrogel composition, degradation, mechanics, and spheroid release require optimization	Relatively high
Oxygen-generating and microenvironment-modulating biomaterials	Temporary oxygen supplementation and/or modulation of the inflammatory niche	↑ early cell survival, ↓ apoptosis, improved angiogenesis and osteogenesis	Oxygen-releasing CaO_2_ system improved MSC survival and ONFH repair [6]; Immunomodulatory hydrogel improved the ONFH niche [65]	Excessive O_2_ may increase ROS; studies did not directly use MSC spheroids; increased material complexity	Moderate/preclinical
Controlled delivery of angiogenic and osteogenic factors	Early VEGF/FGF-2 signaling followed by sustained osteogenic signaling such as BMPs	Restoration of vascular supply followed by enhanced bone formation	Local FGF-2 hydrogel therapy has clinical evidence in precollapse ONFH [66,67,68]	MSC spheroid + FGF-2/VEGF/BMP combination has not been clinically validated; dose and release kinetics must be optimized	Moderate–high
MSC–endothelial co-spheroids/prevascularization	Direct interaction between MSCs and endothelial cells; accelerated microvascular-network formation	Rapid neovascularization and enhanced angiogenesis–osteogenesis coupling	MSC/endothelial co-transplantation improved ONFH repair [69]; Spatially organized MSC spheroids enhanced therapeutic angiogenesis [70]	Additional cell type increases manufacturing, safety, and regulatory complexity	Moderate/preclinical
Bioactive and mechanically supportive scaffolds	Structural support combined with osteoconductive, angiogenic and cell-protective signals	Maintenance of femoral-head architecture and enhanced vascularized bone regeneration	BM-MSC-loaded 3D microspheres + bioactive Mg scaffold enhanced repair in rabbit ONFH [71]	More invasive; complex manufacturing; most relevant to larger lesions; not yet validated with true MSC spheroids	Preclinical

Note: ↑ and ↓ indicate increased and decreased responses.

## Data Availability

No new data were created or analyzed in this study. Data sharing is not applicable to this article.
